# Tenant Reports of In-Home Asthma Triggers and Adult Emergency Department Use

**DOI:** 10.1001/jamanetworkopen.2025.37874

**Published:** 2025-10-16

**Authors:** Zichuan Li, Sophia S. Carryl, Elizabeth A. Samuels, Dinah Foer, Adam L. Haber

**Affiliations:** 1 Department of Environmental Health, Harvard T.H. Chan School of Public Health, Boston, Massachusetts; 2 Department of Emergency Medicine, David Geffen School of Medicine, University of California, Los Angeles; 3 Divisions of Allergy and Clinical Immunology and General Internal Medicine and Primary Care, Department of Medicine, Brigham and Women’s Hospital, Boston, Massachusetts; 4 Harvard Medical School, Boston, Massachusetts

## Abstract

**Importance:**

Housing conditions are well recognized as a significant contributor to asthma burden. However, due to the inherent difficulty of in-home exposure assessment, there is only limited evidence evaluating the extent of population-level exposure to indoor triggers in urban environments and its link with asthma burden and disparities.

**Objective:**

To test the association between the rate of tenant reports of in-home asthma triggers and the population-based incidence rate of adult asthma emergency department (ED) visits in Boston, Massachusetts.

**Design, Setting, and Participants:**

In this cross-sectional study, tenant reports of in-home asthma triggers were obtained from Boston’s housing code enforcement system. Adult asthma ED visits for Boston residents were obtained from a large health system’s electronic records. Tenant reports and ED data from 2021 to 2024 and 2014 to 2018 were aggregated at the census block group level. Data from 2014 to 2018 were used as a sensitivity analysis. Main analyses were conducted January 2021 to December 2024, with a sensitivity analysis conducted January 2014 to December 2018.

**Exposure:**

Block group–level in-home asthma trigger rates across Boston were constructed using housing reporting data and demographic information from the American Community Survey.

**Main Outcomes and Measures:**

The primary outcome was the block group rate of adult asthma ED visits. Associations were tested using a bayesian generalized linear mixed model to account for spatial autocorrelation, block group demographics, household income, and traffic density.

**Results:**

In the main analysis, 2406 asthma ED visits from 1698 unique patients (median [IQR] age, 40.0 [28.0-58.0] years; 1170 female [68.9%]; 29 Asian [1.7%], 627 Black [36.9%], 271 Hispanic [16.0%], 421 White [24.8%], 621 other [36.6%]), and 7259 tenant reports linked to 552 residential block groups were analyzed. The median (IQR) rate of tenant reports at the block group level was 3.98 (1.89-6.74) per 1000 tenant-years and of adult asthma ED visits was 6.34 (2.43-16.08) per 10 000 person-years. In adjusted models, for a 1-IQR increase in the tenant report rate, there was an 8.6% (95% CI, 2.3%-16.2%) increase in the asthma ED visit rate. A sensitivity analysis of a secondary cohort including 4082 visits and 2753 patients confirmed the association between the tenant-reported asthma triggers and adult asthma ED visits, finding a 25.0% increase (95% CI, 16.9%-34.1%).

**Conclusions and Relevance:**

In this cross-sectional study, tenant-reported asthma triggers were found to be associated with adult asthma ED visits after adjusting for neighborhood-level confounders and spatial autocorrelation. These findings underscore the urgent need for policies to improve housing conditions to reduce neighborhood asthma burden.

## Key Points

**Question:**

Is there an association between the neighborhood rate of tenant reports of in-home asthma triggers and adult asthma emergency department (ED) usage?

**Findings:**

In this cross-sectional study including 2406 asthma ED visits from 1698 patients, tenant-reported residential asthma triggers were significantly associated with adult asthma ED visit rates in Boston after adjusting for neighborhood characteristics and spatial autocorrelation.

**Meaning:**

These results suggest that the continuing contribution of unhealthy housing to adult asthma burden and disparities requires renewed efforts to improve code enforcement and universal access to healthy housing.

## Teaser

This cross-sectional study of US adults treated in a large health system examines the association of tenant-reported residential asthma triggers with adult asthma emergency department visits.

## Introduction

Asthma is a chronic lung disease characterized by shortness of breath, wheezing, persistent airway inflammation, and occasional exacerbations or attacks that can be life threatening. In the US, 7.9% of adults report current asthma, with the highest prevalence (8.8%) in the Northeast region.^1^ Asthma exacerbations are a primary driver of morbidity and mortality. Despite advances in treatment, 10.1% of adults with asthma report at least 1 emergency department (ED) visit for asthma annually.^1^ The neighborhood context, particularly housing conditions, has emerged as a significant feature underlying asthma risk.^2,3^ Indoor temperature and mold or dampness is significantly associated with poor asthma control in adults.^4^ Together, these findings have prompted a renewed focus on housing conditions and the effects of remediation on asthma outcomes.^5,6,7^ Additionally, there is continued focus on analyzing mechanisms through which social inequality and structural racism affect access to healthy housing and thus contribute to disparities in asthma prevalence and outcomes.^8,9^

While the causal association between unhealthy housing conditions and asthma outcomes has been well studied at the individual level (cohort or case-control designs), the extent to which those exposures drive asthma burden at the population level, particularly in adults, is unclear.^10,11,12^ This gap limits our knowledge of the extent and dynamics of relevant indoor exposures and hinders our ability to estimate the potential benefits of interventions on adult asthma outcomes, which require robust effect estimates at the population level to inform policy. The complex architecture of cities and the inherently private nature of indoor living spaces complicate spatial and temporal assessments of asthma-trigger exposure patterns and their relationship to asthma. Beck and colleagues addressed this challenge by using housing code violations at the census tract level to identify an association with childhood asthma ED visits in Cincinnati, Ohio,^13^ demonstrating that routinely collected administrative data can provide a critical proxy of indoor exposure patterns citywide.

Many cities in the US use housing codes as a legal mechanism to protect resident health. Code enforcement inspections, which can identify violations, are primarily prompted by tenant reports of in-home violations (including asthma triggers such as mold or rodents). As a result, tenant reports typically occur upstream of code violations and offer a more comprehensive, citywide indicator of patterns in indoor exposures to asthma triggers.

Tenant reports are therefore a unique dataset that can facilitate population-level study of the interrelationships between housing conditions, asthma, and health disparities. In the city of Boston, Massachusetts, asthma prevalence is 25.9% higher among Black residents than White residents, and the annual rate of asthma ED visits for Black residents and Latino residents is respectively 9.0-fold and 4.4-fold higher than for White residents.^14^ Asthma prevalence is 37.8% higher for all adults living in households with an annual income of less than $25 000 compared with adults living in households with an annual income of $50 000 or higher.^14^

While such disparities in asthma prevalence and burden are well recognized, the underlying environmental exposures have received less attention. Our 2022 study of tenant reports in Boston^15^ found that neighborhoods with either lower proportions of White residents or lower median household incomes reported a higher incidence of in-home asthma triggers. However, there are no studies that have examined the association between in-home asthma triggers and acute care utilization for adult asthma at the population level. In this retrospective cross-sectional study, we analyze administrative data and electronic health records to quantify the association between tenant reports of in-home asthma triggers and adult asthma ED visits at the census block group level.

## Methods

### Study Setting and Population

Boston has approximately 663 972 residents across 580 census block groups according to the American Community Survey (2019-2023). In 2021, approximately 13.1% of Boston’s adult residents had asthma, compared with 11.7% of Massachusetts adults and 9.8% of adults nationwide, and approximately 43.6 annual asthma ED visits per 10 000 residents.^14^ As previously noted, there is a higher prevalence of adult asthma and asthma ED visits among Bostonians who are Black, Hispanic or Latino, and/or living in households with an annual income of less than $25 000.

The hospitals in this study are part of the Massachusetts General Brigham health system, which provides adult emergency care at locations including Brigham and Women’s Hospital, Massachusetts General Hospital, and Brigham and Women’s Faulkner Hospital. Together, these EDs see over 194 000 annual visits.^16,17^ This study followed the Strengthening the Reporting of Observational Studies in Epidemiology (STROBE) reporting guideline for cross-sectional studies. This study was approved by the institutional review board at Harvard University and Massachusetts General Brigham. Informed consent was waived because this research could not practicably be carried out without the waiver and posed minimal risk; all data were fully deidentified prior to analysis.

### Asthma Emergency Department Visits

We extracted ED visit data from patients aged 18 years or older with an asthma ED visit between January 1, 2021, and December 31, 2024, from Massachusetts General Brigham’s electronic health records (EHR). To align with available tenant reporting data, patients were included in the analysis if the home address associated with their ED encounter was located within Boston’s geographic boundary.

Encounter-related demographic, diagnosis, and medication data were extracted from the EHR. We defined 2406 asthma-related ED visits with valid addresses in the residential block groups in Boston by those having the appropriate *International Classification of Diseases, Ninth Revision (ICD-9)* and *International Statistical Classification of Diseases and Related Health Problems, Tenth Revision (ICD-10)* diagnostic codes (eTable 1 in Supplement 1) in the primary diagnosis position, and validated that asthma exacerbation medications were administered in the majority of these visits (eTable 2 in Supplement 1). Including asthma symptoms as well as *ICD* codes resulted in more visits (5319 total visits) but a lower rate of medications from 88.6% to 64.4%, and so the expanded criterion was not used further (eTable 2 in Supplement 1).

### Tenant-Reported Unhealthy Housing Conditions

Tenant reports of in-home asthma triggers, defined as asthma-related requests for home inspections by a code enforcement officer, were obtained from Boston’s open data hub.^18^ We identified 8 categories of tenant reports as relevant to asthma based on our previous study,^15^ including: *heat—excessive insufficient*, *pest infestation—residential*, *mice infestation—residential*, *bed bugs*, *chronic dampness/mold*, *Breathe Easy*, *poor ventilation*, and *rodent activity*. Breathe Easy refers to a program that enables clinicians to report asthma triggers to the Inspectional Services Department directly.^19^

To validate that the tenant reports are indeed a valid assessment of indoor exposure to unhealthy housing, we tested the census tract rate of tenant reports of in-home asthma triggers between 2021 to 2024 against the recently defined Housing Quality Metric derived from the 2021 American Housing Survey,^20^ and the result showed a robust and significant correlation (Spearman ρ = 0.54; *P* < .001) (eFigure 1 in Supplement 1).

### Statistical Analysis

We tested for associations between the incidence rate of tenant reports of in-home asthma triggers and the population-based incidence rate of adult asthma ED visits at block group level using 2 steps. Briefly, we first fitted a baseline model (generalized linear model) and checked for significant any spatial autocorrelation and zero-inflation in the residuals. If significant spatial autocorrelation exists, we then fit 2 models accounting for spatial autocorrelation: a bayesian generalized linear mixed model with a random intercept term for the census tract identifier for each block group and a bayesian generalized additive model where a 2-dimensional penalized cubic spline term was fit to the centroid coordinates of each block group. All 3 regression models were fitted with negative-binomial distribution and adjusted for the block group proportion of the population by race and ethnicity, median household income, and traffic proximity and volume indicator as a proxy for traffic density. The proportion of the population by race and ethnicity was calculated using the American Community Survey (2019-2023) from US Census Bureau, and included residents reporting race or ethnicity as Asian, Black or African American, Hispanic or Latino, White, or other (American Indian and Alaska Native, Native Hawaiian and other Pacific Islander, 2 or more categories, or other).^21^ These covariates were determined using a directed acyclic graph (eFigure 2 in Supplement 1) informed by prior studies.^8,22,23^ The results are reported as rate ratios, and full details of the calculation of incidence rate of tenant reports of in-home asthma triggers and population-based incidence rate of adult asthma ED visits, and covariates used in the models are provided in Supplement 1.

Three sensitivity analyses were performed to test the robustness of the main analysis. As the generalized linear model (baseline model) intentionally did not include an adjustment for spatial considerations, we re-fit it to a subset of the data, including only block groups close to the ED locations. This was designed to examine the effect of any potential spatial sampling bias of the asthma ED outcomes and test the strength of the associations. Block groups within 1, 2, 3 miles, 2 miles but not 1 mile, and 3 miles but not 1 mile of study hospitals were included. As a second sensitivity analysis, we re-fit the generalized linear mixed model (primary model) to only the 88.6% of adult asthma ED visits that were linked to asthma medication, to examine the robustness of the definition of asthma ED visits. Lastly, to validate the observed association in a secondary dataset, we added another sensitivity analysis by fitting the model using data from 2014 to 2018.

A Moran *I* test was used to test spatial autocorrelation, and a permutation-based zero-inflation test from the DHARMa package in R version 4.3.3 (R Project for Statistical Computing) was used to test for zero-inflation.^24^ We report 1-sided *P* values for these 2 tests; a result was considered as significant if 1-sided *P* < .05. The effect size estimates were considered significant if the 95% confidence interval or 95% credible interval does not include the null value. Additional details about statistical analysis are provided in Supplement 1.

## Results

### Study Population and Exposure Assessment

The Massachusetts General Brigham’s EHR data between January 1, 2021, and December 31, 2024, included 13 338 adult asthma ED visits (8063 patients) that had valid addresses and met the inclusion criteria for our primary definition of an ED asthma visit. Of these, 10 932 visits outside of residential block groups in Boston were excluded. A total of 2406 visits from 1698 unique Boston resident patients (1170 female [68.9%]; median [IQR] age, 40.0 [28.0-58.0] years) was included in this study. At the majority of these visits (2132 [88.6%]), an asthma rescue medication was given within 24 hours of ED presentation (eTable 2 in Supplement 1). The primary definition of an ED asthma visit was used for all study analyses.

There are 580 block groups in Boston. After exclusion of 28 nonresidential block groups, we retained 552 residential block groups, covering an average of 653 957 residents from 2019 to 2023. Within the 552 residential block groups, 553 544 residents (84.7%) were 18 years of age or older (ie, adult residents) and 385 264 (58.9%) were renters; 65 443 residents (10.0%) were Asian, 141 755 (21.7%) were Black or African American, 125 019 (19.1%) were Hispanic or Latino, 310 268 (47.4%) were White, and 136 491 (20.9%) were in another category (Table 1).

**Table 1.** Features of Study Population, Exposure, Outcome, and Covariate Data for Residents in Boston From 2021 to 2024

Characteristic	Total No. (%) (N = 653 957)	Block group, median (range) [IQR]
Total population, No.^a^	653 957	1084 (30-3680) [791-1442]
Adult residents, No.^a^	553 544 (84.7)	921 (30-3592) [666-1223]
Tenants, No.^a^	385 264 (58.9)	614 (17-2860) [366-924]
Tenant reports of in-home asthma triggers, No. per 1000 tenant-years	7259	3.98 (0-176.65) [1.89-6.74]
Adult asthma ED visits, No. per 10 000 person-years^b^	2406	6.34 (0-224.19) [2.43-16.08]
Imputed median household income, $^a^	NA	99 600 (5400-250 000) [63 200-143 600]
Traffic proximity and volume indicator, 100 count/km^c^	NA	8.03 (0.29-143.51) [3.05-28.23]
Race or ethnicity, %^a^		
Asian	65 443 (10.0)	5.2 (0-92.6) [0.9-15.2]
Black or African American	141 755 (21.7)	8.5 (0-100.) [2.0-37.5]
Hispanic or Latino	125 019 (19.1)	12.4 (0-90) [5.2-25.9]
White	310 268 (47.4)	52.9 (0-100) [21.5-74.7]
Other^d^	136 491 (20.9)	14.8 (0-90.0) [7.8-28.8]

Abbreviations: ED, emergency department; NA, not applicable.

^a^ 2019-2023 American Community Survey.

^b^ Massachusetts General Health Brigham system hospitals in Boston.

^c^ US Environmental Protection Agency Environmental Justice Screening and Mapping Tool.

^d^ Other includes American Indian and Alaska Native, Native Hawaiian and other Pacific Islander, races that do not belong to mentioned categories, and 2 or more races.

A total of 7259 tenant reports of in-home asthma triggers met inclusion for analysis. The median (IQR) incidence rate of tenant reports of in-home asthma triggers was 3.98 (1.89-6.74) per 1000 tenant-years. The corresponding population-based incidence rate of adult asthma ED visits was 6.34 (2.43-16.08) per 10 000 person-years (Table 1).

### Spatial Distribution and Exposure Disparities

The spatial distribution of ED visits and tenant reports across Boston is shown in Figure. The spatial pattern of asthma ED visits showed qualitative overlap with the distribution of tenant reports of in-home asthma triggers. The rate of tenant reports was substantially higher in Black and Latino communities: median report rates were 172% higher in block groups with the highest quartile of Black or African American residents (6.39 reports per 1000 tenant-years) compared with the lowest (2.35 reports per 1000 tenant-years) and 72% higher comparing the highest proportion of Hispanic or Latino residents (5.16 reports per 1000 tenant-years) with the lowest proportion (3.00 reports per 1000 tenant-years) (eTable 3 in Supplement 1).

**Figure.**
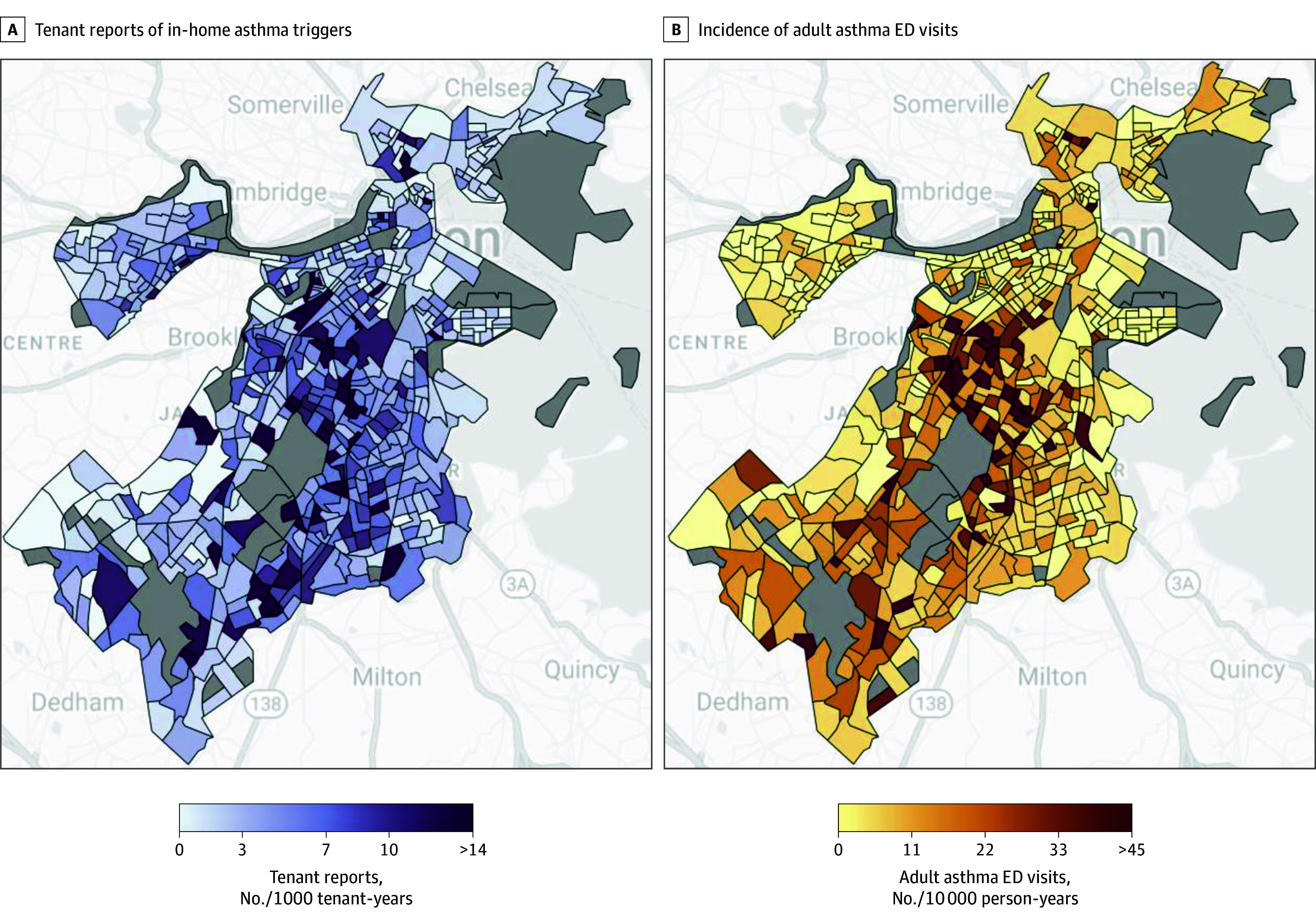
Geospatial Patterns of Tenant Reports of In-Home Asthma Triggers and Adult Asthma Emergency Department (ED) Visits in Boston Choropleth maps of the incidence rate of tenant reports of in-home asthma triggers (left) and the population-based incidence rate of adult asthma ED visits (right) for residential census block groups in Boston. Nonresidential census block groups (gray) were excluded from the analysis.

### Association Between Tenant-Reported Asthma Triggers and Asthma ED Visits

We identified significant spatial autocorrelation (*P* = .001) but not zero-inflation (*P* = .76) in the residuals of the baseline model (generalized linear model) (eTable 4 in Supplement 1). In accounting for spatial autocorrelation, the primary model (generalized linear mixed model) showed that the rate ratio per 1-IQR increase in the incidence rate of tenant reports of in-home asthma triggers was 1.09 (95% credible interval [CrI], 1.02-1.16) for asthma ED visits (Table 2). Results from the secondary model (generalized additive model) were similar (eTable 4 in Supplement 1). Both primary and secondary models showed no statistically significant spatial autocorrelation of their residuals (*P* = .84 and *P* = .81, respectively), indicating that both models addressed the spatial autocorrelation found in the baseline model (Table 2; eTable 4 and eFigure 3 in Supplement 1). All models were adjusted for neighborhood-level demographics and traffic density to control for possible confounders. The results across models indicated a robust and significant positive association between the incidence rate of tenant reports of in-home asthma triggers and the population-based incidence rate of asthma ED visits (primary model: IR per 1-IQR increase, 1.09; 95% CrI, 1.02-1.16). In all models, we observed that the proportion of Black or African American residents (primary model: RR per 10%, 1.16; 95% CrI, 1.11-1.23) and Hispanic or Latino residents (primary model: RR per 10%, 1.14; 95% CrI, 1.07-1.22) were both significantly positively associated with higher adult asthma ED visit rates after adjusting for tenant reports and other covariates (Table 2; eTable 4 in Supplement 1).

**Table 2.** Association Between Tenant Reports of In-Home Asthma Triggers and Adult Asthma Emergency Department Visits Assessed Using a Generalized Linear Mixed Model (Primary Model)^a^

Measure	Adult asthma ED visits, RR (95% CrI)
Tenant reports of in-home asthma triggers (per IQR), IR (95% CI)	1.09 (1.02-1.16)
Proportion of Black or African American population (per 10%)	1.16 (1.11-1.23)
Proportion of Hispanic population (per 10%)	1.14 (1.07-1.22)
Moran *I* test, *P* value	.84

Abbreviations: CrI, credible interval; ED, emergency department; IR, incidence rate; RR, rate ratio.

^a^ IQR of incidence rate of tenant reports of in-home asthma triggers is 4.85 (1.89-6.74) per 1000 tenant-years. The model was also adjusted for proportion of Asian population, median household income, and traffic density and volume.

### Residual Census Tract–Level Variability of ED Visits

The primary model, a generalized linear mixed model with a census tract–level random intercept term, estimated the variability in the baseline census tract–level population-based incidence rate of asthma ED visits. eFigure 4 in Supplement 1 shows the map of estimated random intercepts for census tracts (within which the fitted block groups are located) and the locations of hospitals included in the study. Census tracts near the locations of included hospitals generally had higher random intercepts compared with more distant tracts. The map of the conditional value of the 2-dimensional spline from the secondary model (generalized additive model) showed similar patterns (eFigure 5 in Supplement 1).

### Sensitivity Analyses to Assess the Risk of Spatial Sampling Bias and the Strength of Patient Inclusion Criteria

After restricting the analysis only to include those block groups within a variable radius from the hospitals’ addresses, we found that the positive relationship between the rates of tenant reports and asthma ED visits was consistent across radii and consistent with results from the main analysis (eTable 5 in Supplement 1).

After refitting the models only using ED visits associated with asthma medications (2132 visits), the RR of the population-based incidence rate of asthma ED visits was 1.09 (95% CrI, 1.03-1.17) (eTable 6 in Supplement 1). These results are consistent with the primary model (RR, 1.09; 95% CrI, 1.02-1.16).

### Sensitivity Analysis in a Secondary Validation Cohort

We used tenant reports and ED data from 2014 to 2018 to refit the models, following the same workflow in our main analysis. The secondary cohort included 4082 adult ED visits and 14 425 tenant reports of in-home asthma triggers corresponding to 538 residential block groups. The median (IQR) incidence rate of tenant reports of in-home asthma triggers was 6.29 (3.54-10.65) per 1000 tenant-years. The corresponding population-based incidence rate of adult asthma ED visits was 9.33 (3.43-21.69) per 10 000 person-years. Both rates are higher from 2014 to 2018, ie, 58.3% higher for tenant report rate and 47.2% higher for adult asthma ED visits rate when compared with rates from 2021 to 2024, and using the same statistical modeling approach, also showed significant positive association between tenant reports of in-home asthma triggers vs adult asthma ED visits. Using this secondary dataset from 2014 to 2018, the RR per 1-IQR increase was 1.25 (95% CrI, 1.17-1.34), while using the primary dataset from 2021 to 2024 it was 1.09 (95% CrI, 1.02-1.16).

## Discussion

This study demonstrates an association between tenant reported in-home asthma triggers and adult asthma burden at the population level in Boston. Our results demonstrate an 8.6% increase in the neighborhood rate of adult asthma-related ED visits per 1-IQR increase in the rate of tenant reports of in-home asthma triggers. Asthma trigger report rates were at least 72% higher in neighborhoods with higher proportions of Black or Latino residents. These results account for systemic spatial variation, demographic, and environmental variables that might have otherwise explained population-level associations between housing conditions and the adult asthma ED rate. We further validated the association between tenant reports and adult asthma ED visits in a secondary cohort of asthma patients who visited the ED from 2014 to 2018, and found that the association was also robust, significant, and positive. Together, these findings demonstrate that the ongoing deleterious effects of the well-established causal relationship between unhealthy housing and asthma are detectable at the population level in Boston, stressing the critical need to improve access to healthy housing conditions to improve respiratory disease burden and disparities.

These results also contribute new knowledge to public health research on environmental health and asthma in several additional ways. First, using tenant report data to track housing exposures enables data collection at high geospatial resolution. This approach overcomes previous challenges in identifying a population-level proxy of housing exposures, capturing multiple in-home exposures, and circumvents reliance on inspector-generated reports, which may be subject to bias.^15^ Second, the study design intentionally links neighborhood-level exposures to neighborhood-level asthma outcomes, informing future policy interventions at scale. Third, the models explicitly adjust for spatial autocorrelation to obtain more robust estimates of associations missing from prior studies.^25^

Prior studies have demonstrated associations between specific asthma triggers with adult and pediatric asthma outcomes at the individual level. Kang et al^4^ reported that lower annual average asthma control test scores were significantly associated with indoor temperature and the presence of visible mold or dampness in an adult cohort in Chicago, Illinois. Sinclair et al^26^ similarly showed that higher levels of in-home mold were associated with increased risk of pediatric and adult asthma prevalence among low-income, Hispanic communities in Eastern Coachella Valley, California. A meta-analysis of 7 studies also concluded that increased exposure to indoor fungi was associated with increased asthma exacerbations.^27^ Schoefer et al^28^ found that specific immunoglobulin E antibodies for dust mites were associated with adult asthma. In a more recent longitudinal retrospective observational cohort study, Shigemasa et al^29^ found significant association between dust mite–dominant sensitization pattern and adult-onset asthma. Individual-level evidence linking exposure to lower temperature and cockroach with adult asthma burden has also been reported.^30,31^ Ahluwalia et al^32^ also showed that cockroach and mouse sensitization was associated with childhood asthma burden.

At the neighborhood (population) level, Zárate et al^8^ examined neighborhood characteristics associated with disparities in adult asthma ED visit rates in Austin, Texas, but did not examine the role of housing conditions. Beck et al^13^ used census tract–level density of housing code violations as a predictor of ED visits in pediatric asthma, but did not study adult asthma or examine tenant reports. Our study builds on this research by focusing on adult asthma, examining multiple asthma triggers, and using tenant reports as a readily available population-level marker to demonstrate the scope of the impact of housing conditions on asthma burden. Because many cities collect and make these data publicly available, this analytic approach can be utilized in other urban centers.

It is well established that historically marginalized groups are disproportionately exposed to unhealthy housing conditions and suffer worse asthma outcomes.^1,8^ In the current study, we observed that the Boston neighborhoods with higher exposure to indoor asthma triggers also had higher asthma ED visit rates, consistent with an effect of indoor triggers on adult asthma disparities by race and class. Notably, in models including housing conditions, neighborhood race and ethnicity remained a significant predictor. We interpret this coefficient estimate (secondary effect^33^) as a direct effect of neighborhood race and ethnicity on asthma, after blocking the effect of unhealthy housing, since there are no known unmeasured confounders that drive both neighborhood race and ethnicity and asthma.^33^ We thus conclude that while housing conditions are a strong mediator of asthma disparities by race, they do not fully explain them. Indeed, there are a myriad of other ways in which social inequality and structural racism can predispose people to asthma risk, including psychosocial stress, barriers to care, and mistreatment within the health care system.^23,34^ Critically, facilitating access to healthy housing conditions either by moving, or through repairs—particularly substantive structural upgrades or improvements to reduce inadequate ventilation, dampness and mold, and exposure to pests—not only remediates allergen risks^35,36,37^ but can also improve psychosocial stressors that contribute to worse asthma outcomes.^38^

### Limitations

The study has several limitations. First, because our design is an ecologic study of the association between population-level housing conditions and adult asthma burden, the association cannot be directly extrapolated to estimate risk faced by individual people. Second, our data source was limited to a single health system in Boston. Therefore, the rate of our outcome—adult asthma ED visits—is an underestimate of total neighborhood ED utilization for asthma. However, using several approaches, we confirmed that this limitation does not affect the validity of our conclusion: first, we used a census tract–level random intercept that captured residual variability in the population-based incidence rate of ED visits. We could then visualize and examine census tracts with lower and higher baseline asthma ED visits. As expected, the census tracts closer to the Massachusetts General Brigham hospitals had a higher baseline ED visit rate, and the tracts closer to other health system EDs had lower baseline ED visit rates. This suggests that our primary model effectively captured a degree of uneven spatial sampling. In addition, we performed a sensitivity analysis limiting inclusion to block groups near the health system’s ED locations, reducing potential for any spatial sampling bias. These results were consistent with our primary results, supporting the robustness of the effect estimates for the association between exposure and outcome.

A third limitation was reliance on *ICD-9 *and *ICD-10* coding to identify adult asthma ED visits. While *ICD* codes are commonly used in population-level research, sensitivity and specificity may vary across institutions. To address this, we constructed 2 definitions of adult asthma ED visits and used the more conservative definition of adult asthma ED visits for our analyses. We also performed a sensitivity analysis that only included ED visits associated with asthma-related medications, which represented the majority of our total cohort, consistent with prior electronic health records studies of asthma in the ED.^39^ The results consistently demonstrated an association between housing conditions and asthma ED utilization, supporting our *ICD*-based asthma ED visit definition.

Lastly, tenant reports of in-home asthma triggers do not directly assess indoor exposure to unhealthy conditions. To address this limitation, we used the recently described Housing Quality Metric as an orthogonal, validation dataset,^20^ which showed good agreement and confirmed that tenant reports offer a robust assessment of indoor exposures, information difficult to gather citywide. In addition to the Housing Quality Metric, our previous Boston-based study also showed that the number of tenant reports is significantly negatively correlated with Real Estate Assessment Center physical inspection scores, where a lower score represents worse housing conditions,^15^ supporting the validity of the tenant-reported conditions and the use of these reports as a reliable proxy for housing conditions. Importantly, recent studies by Kontokosta et al^40^ and McLafferty et al^41^ both showed that in affluent neighborhoods residents tend to over report complaints to 311 compared with low-income or socially marginalized neighborhoods. This effect is at least partly driven by increased fear of landlord or state agency retaliation among marginalized tenants.^42^ Even given potential underreporting from the most exposed communities, the observed tenant report rate is still markedly higher in low-income and majority non-White neighborhoods in Boston, and so therefore effect estimates in our study are likely to be conservative.

## Conclusions

Our results use citywide, high spatial resolution tenant-reporting data to identify a significant association between in-home asthma triggers and asthma ED visits in adults, accounting for confounders and spatial autocorrelation. These data indicate that unhealthy housing remains associated with the stark asthma burden differences between neighborhoods across Boston. Redoubled efforts to improve the effectiveness of housing code enforcement and universal access to healthy housing are essential to reduce overall asthma burden and disparities.

## Notes

Supplement 1. **eTable 1**. Keywords and *International Classification of Diseases* (*ICD*) Codes Used to Identify Asthma and Asthma Symptoms **eTable 2**. Comparison Between Adult Asthma Emergency Department (ED) Visits Defined by an Asthma Diagnosis Code in the Primary *ICD* Code Position, and Adult Asthma ED Visits Defined by an Asthma Diagnosis Code *or* Asthma Symptom Code in the Primary Position **eTable 3.** Incidence Rate of Tenant Reports of In-Home Asthma Triggers at Census Block Group Level by Quartiles of Racial and Ethnic Composition in Boston from 2021 to 2024 **eTable 4**. Results of Association Between Tenant Reports of In-Home Asthma Triggers and Adult Asthma Emergency Department (ED) Visits Using Generalized Linear Model and Bayesian Generalized Additive Model **eTable 5**. Results of Sensitivity Analysis Testing the Association Between Tenant Reports of In-Home Asthma Triggers and Adult Asthma Emergency Department (ED) Visits Including Data From Block Groups Near Mass General Brigham Hospitals **eTable 6**. Results of Sensitivity Analysis Testing the Association Between Tenant Reports of In-Home Asthma Triggers and Adult Asthma Emergency Department (ED) Visits Including Only Adult Asthma ED Visits Associated With Asthma Rescue Medication Using a Bayesian Generalized Linear Mixed Model **eFigure 1. **Scatter Plot of the Incidence Rate of Tenant Reports of Asthma Triggers (This Study) vs Prevalence of Inadequate Units Defined From American Housing Survey Microdata for All Census Tracts (Points) in Boston **eFigure 2**. Directed Acyclic Graph **eFigure 3**. Choropleth Maps of the Value of Residuals From Generalized Linear Mixed Model and Generalized Additive Model **eFigure 4**. Choropleth Maps of the Value of Raw Random Intercept Estimates From the Generalized Linear Mixed Model (Primary Model) Used in the Main Analysis for Each Census Tract in Boston **eFigure 5**. Raster Surface Maps of the Estimated Value of the 2-Dimensional Spline Term From the Generalized Additive Model (Secondary Model) Used in the Main Analysis

Supplement 2.
Data Sharing Statement

## References

[1] Pate CA, Zahran HS, Qin X, Johnson C, Hummelman E, Malilay J. Asthma surveillance—United States, 2006-2018. MMWR Surveill Summ. 2021;70(5):1-32. doi:10.15585/mmwr.ss7005a1 34529643 PMC8480992

[2] Krieger J, Higgins DL. Housing and health: time again for public health action. Am J Public Health. 2002;92(5):758-768. doi:10.2105/AJPH.92.5.758 11988443 PMC1447157

[3] Gold DR, Adamkiewicz G, Arshad SH, . NIAID, NIEHS, NHLBI, and MCAN workshop report: the indoor environment and childhood asthma-implications for home environmental intervention in asthma prevention and management. J Allergy Clin Immunol. 2017;140(4):933-949. doi:10.1016/j.jaci.2017.04.024 28502823 PMC5632590

[4] Kang I, McCreery A, Azimi P, . Impacts of residential indoor air quality and environmental risk factors on adult asthma-related health outcomes in Chicago, IL. J Expo Sci Environ Epidemiol. 2023;33(3):358-367. doi:10.1038/s41370-022-00503-z 36450925

[5] Dorsey C. It takes a village: why community organizing is more effective than litigation alone at ending discriminatory housing code enforcement. Georgetown J Poverty Law Policy. 2005;12(3):437. Accessed April 25, 2025. https://heinonline.org/HOL/LandingPage?handle=hein.journals/geojpovlp12&div=24&id=&page=

[6] Tilburg WC. Policy approaches to improving housing and health. J Law Med Ethics. 2017;45(1_suppl)(suppl):90-93. doi:10.1177/1073110517703334 28661307

[7] Krieger JK, Takaro TK, Allen C, . The Seattle-King County healthy homes project: implementation of a comprehensive approach to improving indoor environmental quality for low-income children with asthma. Environ Health Perspect. 2002;110(Suppl 2)(suppl 2):311-322. doi:10.1289/ehp.02110s2311 11929743 PMC1241178

[8] Zárate RA, Bhavnani D, Chambliss S, . Neighborhood-level variability in asthma-related emergency department visits in Central Texas. J Allergy Clin Immunol. 2024;154(4):933-939. doi:10.1016/j.jaci.2024.05.024 38851399 PMC12285651

[9] Martinez A, de la Rosa R, Mujahid M, Thakur N. Structural racism and its pathways to asthma and atopic dermatitis. J Allergy Clin Immunol. 2021;148(5):1112-1120. doi:10.1016/j.jaci.2021.09.020 34743832 PMC9186508

[10] Northridge J, Ramirez OF, Stingone JA, Claudio L. The role of housing type and housing quality in urban children with asthma. J Urban Health. 2010;87(2):211-224. doi:10.1007/s11524-009-9404-1 20063071 PMC2845835

[11] Holt EW, Theall KP, Rabito FA. Individual, housing, and neighborhood correlates of asthma among young urban children. J Urban Health. 2013;90(1):116-129. doi:10.1007/s11524-012-9709-3 22689297 PMC3579308

[12] Hughes K, Bellis MA, Hardcastle KA, . The effect of multiple adverse childhood experiences on health: a systematic review and meta-analysis. Lancet Public Health. 2017;2(8):e356-e366. doi:10.1016/S2468-2667(17)30118-4 29253477

[13] Beck AF, Huang B, Chundur R, Kahn RS. Housing code violation density associated with emergency department and hospital use by children with asthma. Health Aff (Millwood). 2014;33(11):1993-2002. doi:10.1377/hlthaff.2014.0496 25367995 PMC4458371

[14] Boston Public Health Commission. Health of Boston 2023: the asthma report. Published online 2023. Accessed April 25, 2025. https://www.boston.gov/sites/default/files/file/2023/05/HOB_Asthma_2023_FINAL_May11.pdf

[15] Lemire E, Samuels EA, Wang W, Haber A. Unequal housing conditions and code enforcement contribute to asthma disparities in Boston, Massachusetts. Health Aff (Millwood). 2022;41(4):563-572. doi:10.1377/hlthaff.2021.01403 35377754 PMC11901997

[16] Department of Emergency Medicine–Brigham and Women’s Hospital. Accessed November 21, 2024. https://www.brighamandwomens.org/emergency-medicine

[17] Harvard Affiliated Emergency Medicine Residency. Partner organizations. Accessed November 21, 2024. https://haemr.org/residency/clinical-training-sites/

[18] Analyze Boston. 311 Service Requests. Accessed November 21, 2024. https://data.boston.gov/dataset/311-service-requests

[19] Reid M, Fiffer M, Gunturi N, Ali A, Irish D, Sandel M. Breathe easy at home: a web-based referral system linking clinical sites with housing code enforcement for patients with asthma. J Environ Health. 2014;76(7):36-39. 24683937

[20] Garrison V, Ashley PJ, Moran AJ, Cudjoe TKM, Perrin EM, Pollack CE. Housing quality metric (HQM): neighborhood-level data, housing quality, and population health. Am J Public Health. 2025;115(5):780-788. doi:10.2105/AJPH.2024.307962 39982416 PMC11983048

[21] Bureau UC. Data Releases. Census.gov. Accessed July 29, 2025. https://www.census.gov/programs-surveys/acs/news/data-releases.html

[22] Zhang AM, Banzon TM, Phipatanakul W. The spectrum of environmental disparities in asthma. J Allergy Clin Immunol. 2024;153(2):398-400. doi:10.1016/j.jaci.2023.09.004 37717627 PMC11332658

[23] Grant T, Croce E, Matsui EC. Asthma and the social determinants of health. Ann Allergy Asthma Immunol. 2022;128(1):5-11. doi:10.1016/j.anai.2021.10.002 34673220 PMC8671352

[24] Hartig F. DHARMa: residual diagnostics for hierarchical (multi-level/mixed) regression models. 2024. https://github.com/florianhartig/dharma

[25] DeMass R, Gupta D, Self S, Thomas D, Rudisill C. Emergency department use and geospatial variation in social determinants of health: a pilot study from South Carolina. BMC Public Health. 2023;23(1):1527. doi:10.1186/s12889-023-16136-2 37563566 PMC10416539

[26] Sinclair R, Russell C, Kray G, Vesper S. Asthma risk associated with indoor mold contamination in Hispanic communities in Eastern Coachella Valley, California. J Environ Public Health. 2018;2018:9350370. doi:10.1155/2018/9350370 30410546 PMC6205096

[27] Sharpe RA, Bearman N, Thornton CR, Husk K, Osborne NJ. Indoor fungal diversity and asthma: a meta-analysis and systematic review of risk factors. J Allergy Clin Immunol. 2015;135(1):110-122. doi:10.1016/j.jaci.2014.07.002 25159468

[28] Schoefer Y, Schäfer T, Meisinger C, Wichmann HE, Heinrich J; KORA study group. Predictivity of allergic sensitization (RAST) for the onset of allergic diseases in adults. Allergy. 2008;63(1):81-86. doi:10.1111/j.1398-9995.2007.01517.x 18053017

[29] Shigemasa R, Masuko H, Oshima H, . Dust mite-dominant sensitization pattern as a causal factor for adult-onset asthma. Allergol Int. 2021;70(3):368-369. doi:10.1016/j.alit.2021.02.004 33762158

[30] Kang BC, Johnson J, Veres-Thorner C. Atopic profile of inner-city asthma with a comparative analysis on the cockroach-sensitive and ragweed-sensitive subgroups. J Allergy Clin Immunol. 1993;92(6):802-811. doi:10.1016/0091-6749(93)90057-M 8258614

[31] May L, Carim M, Yadav K. Adult asthma exacerbations and environmental triggers: a retrospective review of ED visits using an electronic medical record. Am J Emerg Med. 2011;29(9):1074-1082. doi:10.1016/j.ajem.2010.06.034 20708875

[32] Ahluwalia SK, Peng RD, Breysse PN, . Mouse allergen is the major allergen of public health relevance in Baltimore City. J Allergy Clin Immunol. 2013;132(4):830-835. doi:10.1016/j.jaci.2013.05.005 23810154 PMC3800085

[33] Westreich D, Greenland S. The table 2 fallacy: presenting and interpreting confounder and modifier coefficients. Am J Epidemiol. 2013;177(4):292-298. doi:10.1093/aje/kws412 23371353 PMC3626058

[34] Beck AF, Huang B, Auger KA, Ryan PH, Chen C, Kahn RS. Explaining racial disparities in child asthma readmission using a causal inference approach. JAMA Pediatr. 2016;170(7):695-703. doi:10.1001/jamapediatrics.2016.0269 27182793 PMC5503118

[35] Colton MD, Laurent JGC, MacNaughton P, . Health benefits of green public housing: associations with asthma morbidity and building-related symptoms. Am J Public Health. 2015;105(12):2482-2489. doi:10.2105/AJPH.2015.302793 26469661 PMC4638234

[36] Bryant-Stephens TC, Strane D, Robinson EK, Bhambhani S, Kenyon CC. Housing and asthma disparities. J Allergy Clin Immunol. 2021;148(5):1121-1129. doi:10.1016/j.jaci.2021.09.023 34599980 PMC9809049

[37] Beck AF, Wymer L, Pinzer E, Friedman W, Ashley PJ, Vesper S. Reduced prevalence of childhood asthma after housing renovations in an underresourced community. J Allergy Clin Immunol Glob. 2023;2(4):1-4. doi:10.1016/j.jacig.2023.100143 37680344 PMC10481638

[38] Pollack CE, Roberts LC, Peng RD, . Association of a housing mobility program with childhood asthma symptoms and exacerbations. JAMA. 2023;329(19):1671-1681. doi:10.1001/jama.2023.6488 37191703 PMC10189571

[39] Aguilar R, Knudsen-Robbins C, Ehwerhemuepha L, Feaster W, Kamath S, Heyming TW. Pediatric asthma exacerbations: 14-day emergency department return visit risk factors. J Emerg Med. 2024;67(1):e22-e30. doi:10.1016/j.jemermed.2024.02.002 38824038

[40] McLafferty S, Schneider D, Abelt K. Placing volunteered geographic health information: socio-spatial bias in 311 bed bug report data for New York City. Health Place. 2020;62:102282. doi:10.1016/j.healthplace.2019.102282 32479360

[41] Kontokosta CE, Hong B. Bias in smart city governance: How socio-spatial disparities in 311 complaint behavior impact the fairness of data-driven decisions. Sustain Cities Soc. 2021;64:102503. doi:10.1016/j.scs.2020.102503

[42] Rosofsky A, Reid M, Sandel M, Zielenbach M, Murphy J, Scammell MK. Breathe easy at home: a qualitative evaluation of a pediatric asthma intervention. Glob Qual Nurs Res. 2016;3:2333393616676154. doi:10.1177/2333393616676154 28462348 PMC5342293

