# Supplementary material for: Tenant Reports of In-Home Asthma Triggers and Adult Emergency Department Use
Source: JAMA Netw Open. 2025 Oct 16;8(10):e2537874. doi: 10.1001/jamanetworkopen.2025.37874 (PMC12531877; doi:10.1001/jamanetworkopen.2025.37874)
Supplement: Supplement 1. — eTable 1. Keywords and International Classification of Diseases (ICD) Codes Used to Identify Asthma and Asthma Symptoms eTable 2. Comparison Between Adult Asthma Emergency Department (ED) Visits Defined by an Asthma Diagnosis Code in the Primary ICD Code Position, and Adult Asthma ED Visits Defined by an Asthma Diagnosis Code or Asthma Symptom Code in the Primary Position eTable 3. Incidence Rate of Tenant Reports of In-Home Asthma Triggers at Census Block Group Level by Quartiles of Racial and Ethnic Composition in Boston from 2021 to 2024 eTable 4. Results of Association Between Tenant Reports of In-Home Asthma Triggers and Adult Asthma Emergency Department (ED) Visits Using Generalized Linear Model and Bayesian Generalized Additive Model eTable 5. Results of Sensitivity Analysis Testing the Association Between Tenant Reports of In-Home Asthma Triggers and Adult Asthma Emergency Department (ED) Visits Including Data From Block Groups Near Mass General Brigham Hospitals eTable 6. Results of Sensitivity Analysis Testing the Association Between Tenant Reports of In-Home Asthma Triggers and Adult Asthma Emergency Department (ED) Visits Including Only Adult Asthma ED Visits Associated With Asthma Rescue Medication Using a Bayesian Generalized Linear Mixed Model eFigure 1. Scatter Plot of the Incidence Rate of Tenant Reports of Asthma Triggers (This Study) vs Prevalence of Inadequate Units Defined From American Housing Survey Microdata for All Census Tracts (Points) in Boston eFigure 2. Directed Acyclic Graph eFigure 3. Choropleth Maps of the Value of Residuals From Generalized Linear Mixed Model and Generalized Additive Model eFigure 4. Choropleth Maps of the Value of Raw Random Intercept Estimates From the Generalized Linear Mixed Model (Primary Model) Used in the Main Analysis for Each Census Tract in Boston eFigure 5. Raster Surface Maps of the Estimated Value of the 2-Dimensional Spline Term From the Generalized Additive Model (Secondary Model) Used in the Main Ana [file jamanetwopen-e2537874-s001.pdf]

Supplemental Online Content

Li Z, Carryl SS, Samuels EA, Foer D, Haber AL. Tenant reports of in-home asthma triggers and adult emergency department use. *JAMA Netw Open.* 2025;8(10):e2537874. doi:10.1001/jamanetworkopen.2025.37874

eTable 1. Keywords and *International Classification of Diseases* (*ICD*) Codes Used to Identify Asthma and Asthma Symptoms

eTable 2. Comparison Between Adult Asthma Emergency Department (ED) Visits Defined by an Asthma Diagnosis Code in the Primary *ICD* Code Position, and Adult Asthma ED Visits Defined by an Asthma Diagnosis Code *or* Asthma Symptom Code in the Primary Position

eTable 3. Incidence Rate of Tenant Reports of In-Home Asthma Triggers at Census Block Group Level by Quartiles of Racial and Ethnic Composition in Boston from 2021 to 2024

eTable 4. Results of Association Between Tenant Reports of In-Home Asthma Triggers and Adult Asthma Emergency Department (ED) Visits Using Generalized Linear Model and Bayesian Generalized Additive Model

eTable 5. Results of Sensitivity Analysis Testing the Association Between Tenant Reports of In-Home Asthma Triggers and Adult Asthma Emergency Department (ED) Visits Including Data From Block Groups Near Mass General Brigham Hospitals

eTable 6. Results of Sensitivity Analysis Testing the Association Between Tenant Reports of In-Home Asthma Triggers and Adult Asthma Emergency Department (ED) Visits Including Only Adult Asthma ED Visits Associated With Asthma Rescue Medication Using a Bayesian Generalized Linear Mixed Model

eFigure 1. Scatter Plot of the Incidence Rate of Tenant Reports of Asthma Triggers (This Study) vs Prevalence of Inadequate Units Defined From American Housing Survey Microdata for All Census Tracts (Points) in Boston

eFigure 2. Directed Acyclic Graph

eFigure 3. Choropleth Maps of the Value of Residuals From Generalized Linear Mixed Model and Generalized Additive Model

© 2025 Li Z et al. *JAMA Network Open*.

eFigure 4. Choropleth Maps of the Value of Raw Random Intercept Estimates From the Generalized Linear Mixed Model (Primary Model) Used in the Main Analysis for Each Census Tract in Boston

eFigure 5. Raster Surface Maps of the Estimated Value of the 2-Dimensional Spline Term From the Generalized Additive Model (Secondary Model) Used in the Main Analysis

This supplemental material has been provided by the authors to give readers additional information about their work.

© 2025 Li Z et al. *JAMA Network Open*.

Additional details of statistical analysis

Asthma-related ED visits were defined using two definitions[1,2]: The primary definition was an ED visit with an International Classification of Diseases (ICD) diagnostic code for asthma in the primary coded position (**eTable 1** in the Online Supplement). As many asthma visits to the ED present as a symptom-based complaint, we examined an additional definition as a secondary analysis: ED visits with an asthma diagnosis code *or* asthma symptom code in the primary position. The keywords and codes used to identify asthma are shown in **eTable 1**. We also examined the percentages of visits where asthma exacerbation medications were administered within 24 hours of ED admission (inhaled and nebulized short-acting beta-agonists (SABAs), SABAs in combination with anti-cholinergics, inhaled glucocorticoids, and their combinations with other medications, including oral and intravenous glucocorticoids).

All tenant reports identified as in-home asthma triggers reports filed between January 1, 2021 and December 31, 2024 and linked to valid addresses within Boston were analyzed. Reports label as “invalid”, “duplicated” or “owner occupied” were excluded.

Neighborhood demographics, including race, ethnicity, median household income, age-specific population size, and total number of tenants by census block group, were obtained from the American Community Survey (2019-2023). Census block groups, statistical divisions of census tracts, are generally defined to contain between 600 and 3,000 people[3]. Traffic-associated air pollution data using the Traffic Proximity and Volume indicator were obtained from the U.S. Environmental Protection Agency Environmental Justice Screening and Mapping Tool [4]. We imputed missing values of the block group median household income using the average of neighboring block groups. Non-residential block groups, defined as those with less than ten residents or ten tenants, were excluded from the analysis.

Data were geocoded using ArcGIS Pro (version 3.2.0) with an Esri's StreetMap Premium local locator file provided by the Center for Geographic Analysis at Harvard University.

The block group-level incidence rate of tenant reports of in-home asthma triggers was calculated by dividing the total number of reports by the ‘tenant-years’, the product of the number of tenants in the corresponding block group and the number of years in the study period. The block group-level population-based incidence rate for adult ED visits was calculated by dividing the number of ED visits per block group by the ‘adult resident-years’, the product of the number of adult residents and the number of years in the study period.

We tested for associations between the incidence rate of tenant reports of in-home asthma triggers and the population-based incidence rate of adult asthma ED visits using two steps. First, we fitted a generalized linear model with negative-binomial distribution as a baseline model. We then tested for spatial autocorrelation of the residuals using Moran’s *I* [5,6], and zero-inflation using *DHARMa* packing in R[7]. Since the residuals of the baseline model indeed showed significant spatial autocorrelation, we used two models to account for spatial autocorrelation: 1) a Bayesian

© 2025 Li Z et al. *JAMA Network Open*.

generalized linear mixed model with a random intercept term for the census tract identifier for each block group was fitted. This model assumes that the spatial autocorrelation is due to unmeasured factors clustered at the census tract level and served as the primary model. 2) a Bayesian generalized additive model where a two-dimensional penalized cubic spline term was fit to the centroid coordinates of each block group. This second model assumes that the spatial autocorrelation is due to large-scale *spatially smooth* distributed factors to test the robustness of our assumption about the pattern of spatial autocorrelation[8]. Both models' residuals were then test for spatial autocorrelation using Moran's *I* test.

The equation for generalized linear model (baseline model) is shown below:

$$\log(\mu_i) = x_i^T \beta + \log(E_i)$$

Where the μ_i is the expected value of adult ED visits for block group i; x_i is the covariate vector for block group i, which includes proportion of the Asian residents, proportion of the Black or African American residents, proportion of Hispanic or Latino residents, imputed median household income, and Traffic Proximity and Volume indicator; E_i is the adult resident-years for block group i.

The equation for Bayesian generalized linear mixed model (primary model) is shown below:

$$\log(\mu_i) = x_i^T \beta + b_{ct[i]} + \log(E_i)$$

Where $b_{ct[i]}$ is the random intercept for the census tract that block group i belongs to.

The equation for Bayesian generalized additive model (secondary model) is shown below:

$$\log(\mu_i) = x_i^T \beta + s(lat_i, lon_i) + \log(E_i)$$

Where $s(lat, lon)$ is the two-dimensional penalized cubic spline term fitted to the centroid coordinates of block group i.

Bayesian generalized linear mixed model and Bayesian generalized additive model were fitted using *brms* package in R[9]. For frequentist model (generalized linear model), 95% confidence interval was reported with point estimate, and for Bayesian models (Bayesian generalized linear mixed model and Bayesian generalized additive model), 95% credible interval was reported with point estimate. R package *ggplot2* and *ggmap* were used to make the figures[10,11].

All statistical analysis was performed using the R scientific computing environment (version 4.3.3).

© 2025 Li Z et al. *JAMA Network Open*.

eTable 1: Keywords and International Classification of Diseases (ICD) codes used to identify asthma and asthma symptoms.

	**Keywords**	**ICD codes**
Asthma	asthma	493, J45
Asthma symptoms	shortness of breath, wheezing, dyspnea	786.05, R06.02, 786.07, R06.2, R06.00, R06.09

© 2025 Li Z et al. *JAMA Network Open*.

eTable 2: Comparison between adult asthma emergency department (ED) visits defined by an asthma diagnosis code in the primary ICD code position, and adult asthma ED visits defined by an asthma diagnosis code *or* asthma symptom code in the primary position.

	**With an asthma diagnosis code in the primary position**	**With an asthma diagnosis code *or* asthma symptom code in the primary position**
ED visits, N	2,406	5,319
Patients, N	1,698	2,926
ED visits with asthma exacerbation medications[a] (N, %)	2,132 (88.61%)	3,425 (64.39%)

[a] Albuterol, Levalbuterol, Ipratropium bromide, Ventolin, Proair, Atrovent, Proventil, Xopenex, Methylprednisolone, Prednisone, Prednisolone, Medrol, Solumedrol, Duoneb

© 2025 Li Z et al. *JAMA Network Open*.

eTable 3: Incidence rate of tenant reports of in-home asthma triggers at census block group level by quartiles of racial and ethnic composition in Boston from 2021 to 2024.

	**Quartiles, proportion Black or African American residents**[a]				
	**1st**	**2nd**	**3rd**	**4th**	**Overall**
Number of block groups	138	138	138	138	552
Median (interquartile range): Incidence rate of tenant reports of in-home asthma triggers per 1,000 tenant-year	2.35 (3.15)	2.57 (3.45)	4.85 (4.53)	6.39 (5.44)	3.98 (4.85)
	**Quartiles, proportion Hispanic or Latino residents**[*]				
	**1st**	**2nd**	**3rd**	**4th**	**Overall**
Number of block groups	138	138	138	138	552
Median (interquartile range): Incidence rate of tenant reports of in-home asthma triggers per 1,000 tenant-year	3.00 (4.21)	3.66 (4.22)	4.55 (5.71)	5.16 (4.98)	3.98 (4.85)

[a] 2019-2023 American Community Survey.

© 2025 Li Z et al. *JAMA Network Open*.

eTable 4: Results of association between tenant reports of in-home asthma triggers and adult asthma emergency department (ED) visits using generalized linear model and Bayesian generalized additive model. (Baseline and secondary models for main analysis)

	**Rate ratio of adult asthma ED visits (population-based incidence rate)**	**95% CI**
**Baseline model:** generalized linear model		
IR of tenant reports of in-home asthma triggers (per interquartile range)	1.12	1.04, 1.22
Proportion of Black or African American population (per 10%)	1.15	1.10, 1.20
Proportion of Hispanic population (per 10%)	1.16	1.09, 1.24
Moran's *I* test	*p*<0.01	
Zero-inflation test	*p*=0.76	
**Secondary model:** Bayesian generalized additive model		
IR of tenant reports of in-home asthma triggers (per interquartile range)	1.08	1.02, 1.15
Proportion of Black or African American population (per 10%)	1.11	1.05, 1.18
Proportion of Hispanic population (per 10%)	1.11	1.04, 1.19
Moran's *I* test	*p*=0.81	

IR: incidence rate, 95% CI: 95% confidence interval for generalized linear model and 95% credible interval for Bayesian generalized additive model. The interquartile range of IR of tenant reports of in-home asthma triggers is 4.85 (1.89-6.74) per 1,000 tenant-year; the models were also adjusted for proportion of Asian population, median household income, and Traffic Proximity and Volume indicator.

© 2025 Li Z et al. *JAMA Network Open*.

eTable 5: Results of sensitivity analysis testing the association between tenant reports of in-home asthma triggers and adult asthma emergency department (ED) visits including data from block groups near Mass General Brigham hospitals.

	**Block groups within one mile**	**Block groups within two miles**	**Block groups within three miles**	**Block groups within two miles but not one mile**	**Block groups within three miles but not one mile**
Number of block groups included	141	327	496	186	355
Number of tenant reports of in-home asthma triggers included	1,586	4,254	6,727	2,668	5,141
Number of adult asthma ED visits included	723	1,495	2,202	772	1,479
Rate ratio (95% CI) of adult asthma ED visits per interquartile range increase in exposure	1.15 (0.93, 1.44)	1.17 (1.05, 1.32)	1.11 (1.03, 1.21)	1.15 (1.04, 1.31)	1.08 (1.02, 1.18)

95%CI: 95% confidence interval. The interquartile range of IR of tenant reports of in-home asthma triggers is 4.85 (1.89-6.74) per 1,000 tenant-year.

© 2025 Li Z et al. *JAMA Network Open*.

eTable 6: Results of sensitivity analysis testing the association between tenant reports of in-home asthma triggers and adult asthma emergency department (ED) visits including only adult asthma ED visits associated with asthma rescue medication using a Bayesian generalized linear mixed model.

	**Number of visits**	**Rate ratio per interquartile range increase in tenant reports rate (95%CI)**
Adult asthma ED visits with asthma exacerbation medication	2,132	1.09 (1.03, 1.17)

95%CI=95% credible interval; Interquartile range of IR of tenant reports of in-home asthma triggers is 4.85 (1.89-6.74) per 1,000 tenant-year.

© 2025 Li Z et al. *JAMA Network Open*.

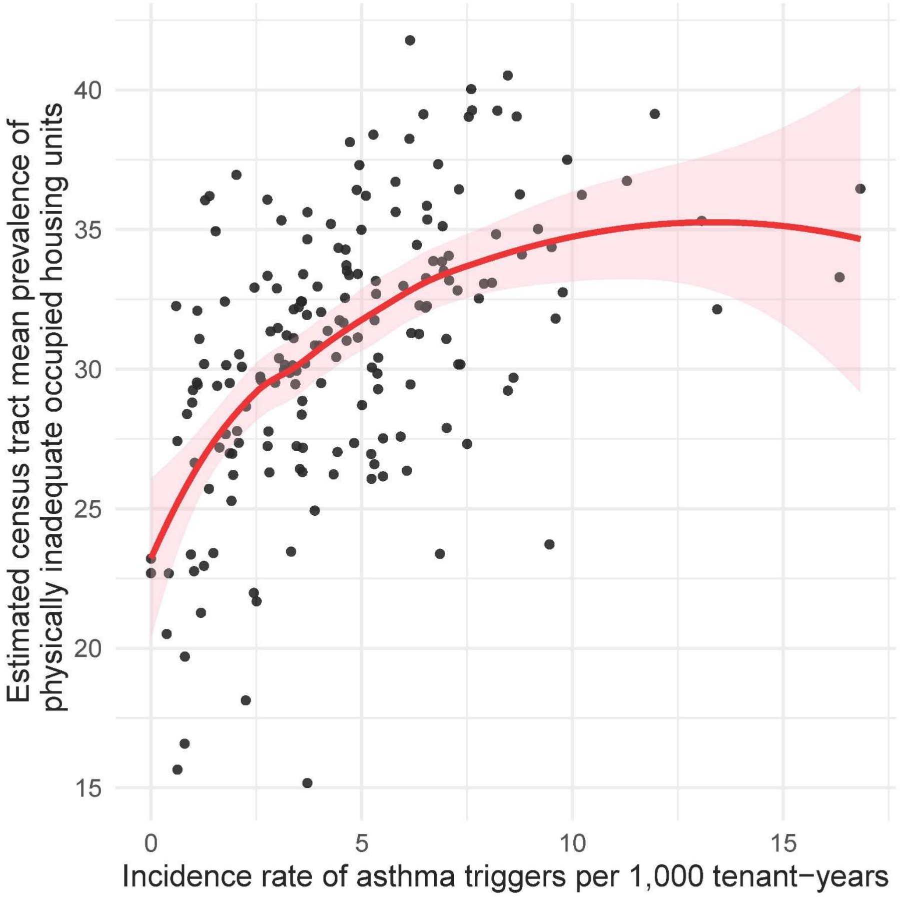

eFigure 1: Scatter plot of the incidence rate of tenant reports of asthma triggers (this study, x axis) vs. prevalence of inadequate units defined from American Housing Survey microdata[12] (y axis) for all census tracts (points) in Boston. Red line: LOESS fit.

© 2025 Li Z et al. *JAMA Network Open*.

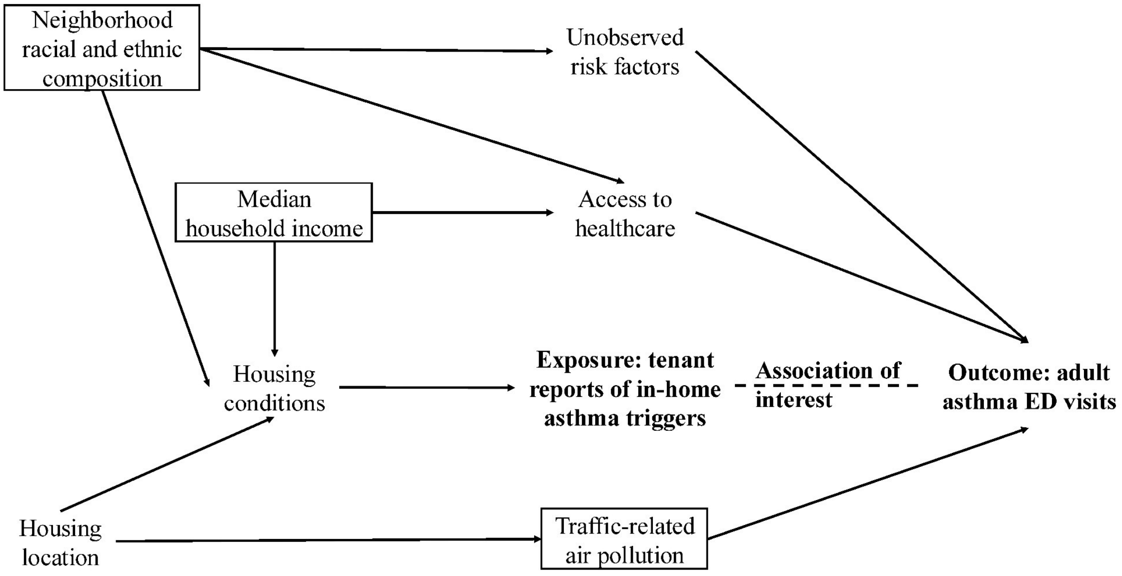

eFigure 2: Directed acyclic graph. Boxed variables were controlled for in all analyses. ED: Emergency Department.

© 2025 Li Z et al. *JAMA Network Open*.

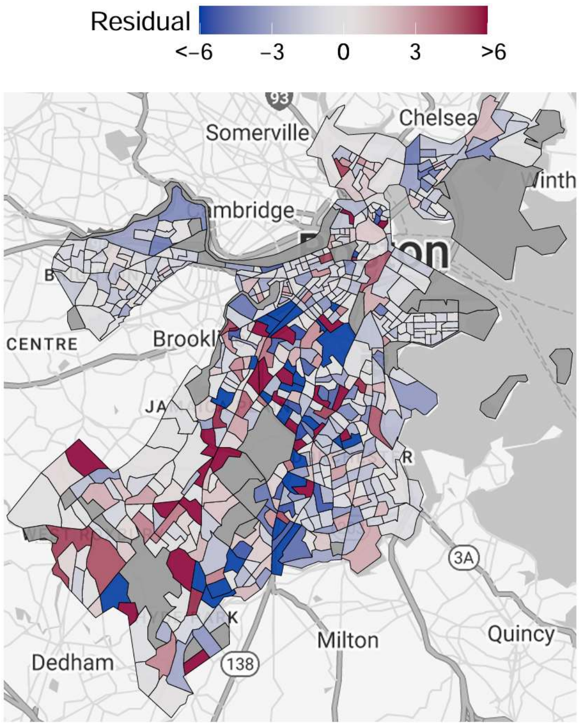

(A)

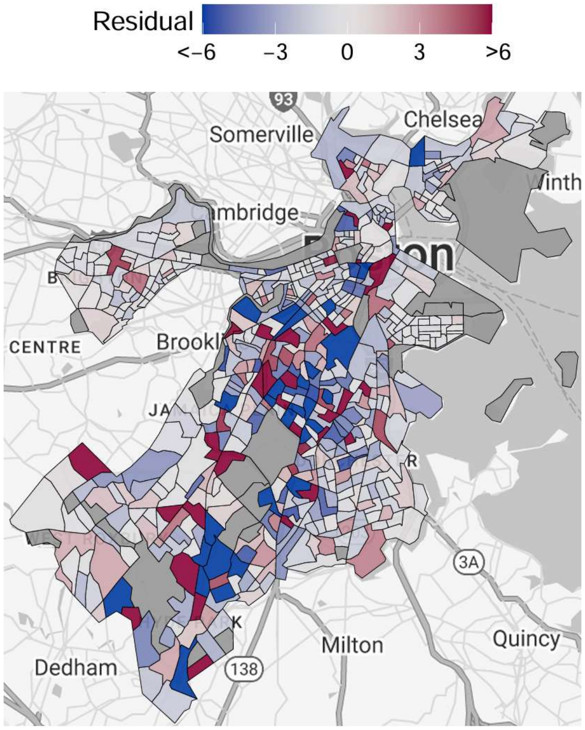

(B)

eFigure 3. Choropleth maps of the value (color legend, top) of residuals from (A) Generalized linear mixed model and (B) Generalized additive model. Non-residential (grey) census block groups were excluded from the analysis.

© 2025 Li Z et al. *JAMA Network Open*.

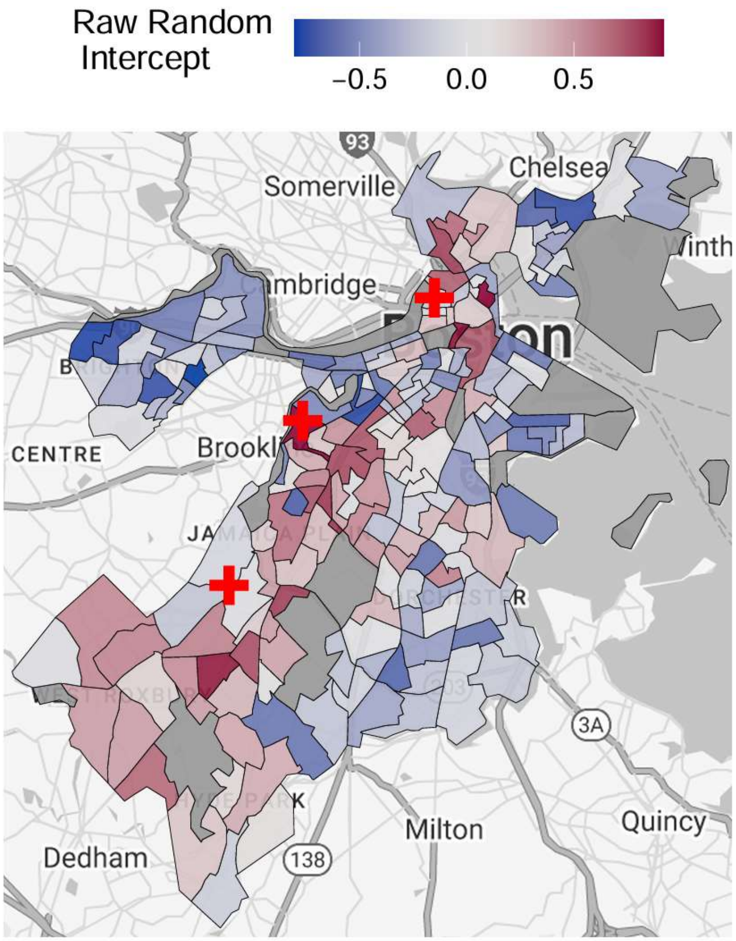

eFigure 4: Choropleth maps of the value (color legend, top) of raw random intercept estimates from the generalized linear mixed model (primary model) used in the main analysis for each census tract in Boston. Red crosses represent locations of Mass General Brigham health system hospitals used in our analysis. Non-residential (grey) census tracts were excluded from the analysis.

© 2025 Li Z et al. *JAMA Network Open*.

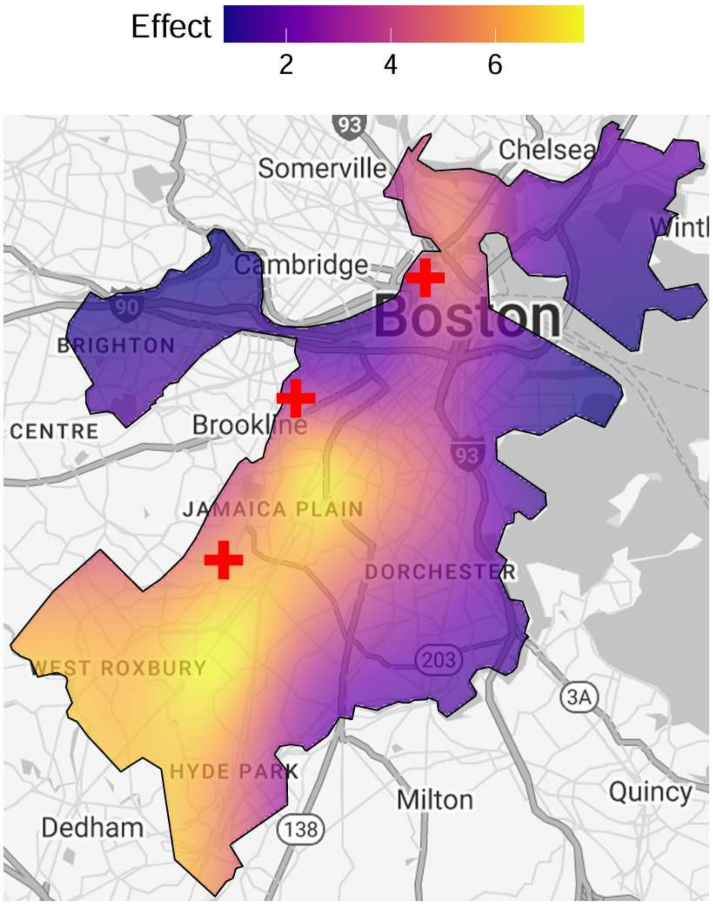

eFigure 5. Raster surface maps of the estimated value of the two-dimensional spline term from the generalized additive model (secondary model) used in the main analysis. Red crosses represent Mass General Brigham health system hospital locations in Boston.

© 2025 Li Z et al. *JAMA Network Open*.

Supplementary References

1. Zárate RA, Bhavnani D, Chambliss S, et al. Neighborhood-level variability in asthma-related emergency department visits in Central Texas. *J Allergy Clin Immunol*. Published online June 6, 2024:S0091-6749(24)00568-2. doi:10.1016/j.jaci.2024.05.024

2. Reddel HK, Taylor DR, Bateman ED, et al. An Official American Thoracic Society/European Respiratory Society Statement: Asthma Control and Exacerbations. *Am J Respir Crit Care Med*. 2009;180(1):59-99. doi:10.1164/rccm.200801-060ST

3. Bureau UC. Glossary. Census.gov. Accessed November 4, 2024. https://www.census.gov/programs-surveys/geography/about/glossary.html

4. US EPA O. EJScreen: Environmental Justice Screening and Mapping Tool. September 3, 2014. Accessed April 10, 2024. https://www.epa.gov/ejscreen

5. Bivand R, Wong DWS. Comparing implementations of global and local indicators of spatial association. *TEST*. 2018;27(3):716-748. doi:10.1007/s11749-018-0599-x

6. Nardone A, Casey JA, Morello-Frosch R, Mujahid M, Balmes JR, Thakur N. Associations between historical residential redlining and current age-adjusted asthma emergency department-visit rates across eight cities of California: an ecological study. *Lancet Planet Health*. 2020;4(1):e24-e31. doi:10.1016/S2542-5196(19)30241-4

7. Hartig F. *DHARMa: Residual Diagnostics for Hierarchical (Multi-Level / Mixed) Regression Models*.; 2024. https://github.com/florianhartig/dharma

8. F. Dormann C, M. McPherson J, B. Araújo M, et al. Methods to account for spatial autocorrelation in the analysis of species distributional data: a review. *Ecography*. 2007;30(5):609-628. doi:10.1111/j.2007.0906-7590.05171.x

9. Bürkner PC. brms: An R Package for Bayesian Multilevel Models Using Stan. *Journal of Statistical Software*. 2017;80:1-28. doi:10.18637/jss.v080.i01

10. Kahle D, Wickham H. ggmap: Spatial Visualization with ggplot2. *The R Journal*. 2013;5(1):144-161.

11. Wickham H. *Ggplot2: Elegant Graphics for Data Analysis*. Springer-Verlag New York; 2016. https://ggplot2.tidyverse.org

12. Garrison V, Ashley PJ, Moran AJ, Cudjoe TKM, Perrin EM, Pollack CE. Housing Quality Metric (HQM): Neighborhood-Level Data, Housing Quality, and Population Health. *Am J Public Health*. Published online February 21, 2025:e1-e9. doi:10.2105/AJPH.2024.307962

© 2025 Li Z et al. *JAMA Network Open*.
