# Supplementary material for: Tenant Reports of In-Home Asthma Triggers and Adult Emergency Department Use
Source: JAMA Netw Open. 2025 Oct 16;8(10):e2537874. doi: 10.1001/jamanetworkopen.2025.37874 (PMC12531877; doi:10.1001/jamanetworkopen.2025.37874)
Supplement: Supplement 2. — Data Sharing Statement [file jamanetwopen-e2537874-s002.pdf]

Data Sharing Statement

Data
Data available: No

Additional Information
Explanation for why data not available: The electronic health records we used in this study can't be made available to others due to the Data Use Agreement. Other data used in this study are all publicly available.
